# Not Just Passing Through: Bone Marrow as a Home for Diverse Resident T Cells

**DOI:** 10.1002/eji.70083

**Published:** 2025-11-10

**Authors:** Kyra D. Zens, Klaas P. J. M. van Gisbergen

**Affiliations:** ^1^ Viral Immunobiology Section, Institute for Experimental Immunology University of Zurich Zurich Switzerland; ^2^ Department of Public and Global Health, Epidemiology, Biostatistics and Prevention Institute University of Zurich Zurich Switzerland; ^3^ Physiology and Cancer Research Programme, Champalimaud Research Champalimaud Foundation Lisbon Portugal

**Keywords:** bone marrow, T cells, tissue‐resident memory

## Abstract

Recent studies add to our understanding of T cell residency in the human bone marrow (BM). In the May 2025 issue of EJI, Schneider Revueltas et al. demonstrate that CD69^−^ CD4^+^ and CD8+ memory T cells, presumed to be recirculating, have a distinct transcriptional cluster profile and a distinct TCR repertoire from their blood counterparts. These findings are in line with CD69^−^ memory T cells taking up residence in the BM, similar to their CD69^+^ counterparts. In parallel, Pulvirenti et al. identify a subset of CD69^+^EOMES^+^GzmK^+^ Tr1‐like cells in the BM maintained by IL‐15. Together, these studies refine our understanding of the BM as a heterogeneous immune niche and suggest a broader definition of resident cells within this tissue.

## Graphical abstract

CD8^+^CD69^−^ memory T cells (Schneider Revueltas et al.) were identified to reside in bone marrow besides canonical CD8^+^CD69^+^ tissue‐resident memory T cells (TRM). In addition, CD4^+^CD69^+^ Tr1‐like cells (Pulvirenti et al.) were preferentially found in bone marrow. Together, these findings illustrate diversity in the T cell subsets that reside long‐term within the bone marrow.

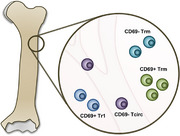

## 1 Introduction: Rethinking T Cell Residency in the Bone Marrow

Until just 15 years ago, memory T cells were thought to circulate continuously throughout the body, with central and effector memory cells passing through secondary lymphoid and peripheral tissues, respectively [1]. Studies in mice demonstrating the persistence of effector‐phenotype cells in barrier tissues long after resolution of infection led to the identification of tissue‐resident memory T cells (TRM) [2, 3]. Since this time, our understanding of TRM has expanded dramatically from initial observations of effector‐like cells in barrier tissues such as the skin, gut, and lung to the more recent realization that phenotypically and functionally similar subsets are also present in nonbarrier niches such as lymphoid tissues and the bone marrow (BM).

The BM has long been recognized as a niche for long‐lived plasma cells (reviewed in [4]) and circulating memory T cells [5, 6], but the presence and functional roles of TRM at this site are less well‐understood. Most studies of TRM have historically identified this subset by expression of the canonical marker CD69, which antagonizes S1PR1‐mediated egress, supporting tissue retention. Studies in both murine and human BM have demonstrated that a large fraction of both CD4^+^ and CD8^+^ memory T cells express CD69, indicating permanent residence at this site [7, 8]. Furthermore, CD69^+^ TRM specific to several common pathogens have been identified in the BM and are maintained in the absence of continued local antigen exposure or inflammation [7, 9]. Yet CD69 is not essential for TRM establishment in all tissues [10]. It remains unclear whether the CD69^−^ memory T cells remain strictly part of the circulation or might also be retained within the tissue in some cases. Furthermore, while T cells specific to many pathogens can be identified within the BM, their functional diversity, and hence their role in immune responses, is poorly understood, as is how such cells are maintained long‐term.

While much remains to be understood about BM TRM, two papers in the May 2025 issue of EJI help to address these questions, offering complementary insights into the diversity of T cell populations that persist within the human BM [11, 12]. Together, these findings highlight the heterogeneity of BM T cells, suggesting that this tissue harbors an even more phenotypically and functionally diverse population of memory T cells than previously appreciated.

## 2 Lack of CD69 May Not Always Mean Circulating: Schneider Revueltas et al

With CD69 likely acting as a key mediator of tissue retention, CD69^−^ memory T cells have generally been considered to be recirculating, poised to exit tissues in response to a sphingosine‐1‐phosphate (S1P) gradient. While, as described, a substantial fraction of CD4^+^ and CD8^+^ memory T cells in the human BM express CD69 on the cell surface, it is also clear that CD69 is not strictly required for the establishment of TRM at all tissue sites [13, 14]. To investigate how CD69^+^ and CD69^−^ BM T cells compare to each other and their counterparts in the blood, Schneider Revueltas et al. [1] used paired BM and blood samples from three human donors, performing CITE‐seq with single‐cell RNA sequencing and TCR repertoire analysis on over 38,000 total CD45RO^+^ memory T cells.

The authors identified nine major transcriptional clusters, each of CD4^+^ and CD8^+^ cells, which differed between blood and BM CD69^+^ and CD69^−^ subsets. In comparing analogous clusters between subsets, TCR repertoire analysis revealed that all three originated from distinct T cell precursors. The limited clonal overlap between blood T cells and CD69^−^ BM T cells suggests that the latter are not part of the recirculating memory pool. However, they also appear distinct from their CD69^+^ counterparts, indicating that they do not form a transitional state between circulating T cells and CD69+ Trm. Interestingly, several populations within these CD69‐ BM T cells transcribed, but did not surface express, CD69 together with S1PR1 and KLF2 that instruct tissue exit. Based on these findings, the authors propose a mechanism involving internalization of the CD69‐S1PR1 complex, which could prevent these cells from leaving the BM niche despite the apparent absence of surface CD69 expression. Other adhesion mechanisms may assist in anchoring CD69^−^ cells in BM, although no evidence has emerged from the transcriptional analysis of differential expression with CD69^+^ BM cells. The transcriptional signature of CD69^−^ BM cells enriched in quiescence‐associated genes, including KLF3, BTG1, and AP‐1, potentially aligns with a long‐lived, tissue‐localized phenotype.

Support for the existence of CD69^−^ Trm comes from mouse studies showing disequilibrium of CD69‐ T cell fractions with the circulating pool in pancreas, kidney, salivary glands, and female reproductive tract [13]. Although further experiments are required to validate the residence of CD69^−^ T cells in BM, the identification of CD69^−^ T cells as Trm expands the conventional view of TRM beyond the canonical CD69^+^ phenotype. An important realization from these studies is that tissue residency in BM and at other sites may involve alternative strategies for local maintenance. Therefore, these findings have implications for understanding how different tissue environments shape TRM phenotypes and for recognizing resident populations that may be overlooked by standard surface‐marker definitions.

## 3 A Niche for Regulation: Pulvirenti et al

In parallel, Pulvirenti et al. [12] investigated a distinct, but complementary, facet of the BM T cell landscape: the potential maintenance of regulatory T cells. The BM not only contains populations of conventional FOXP3^+^ regulatory T cells, but also of type 1 regulatory T cells (Tr1), which are characterized as FOXP3‐independent and IL‐10‐producing CD4 T cells with regulatory function [15]. Previously, IL‐10‐producing, FOXP3^−^ Tr1 have been observed to rapidly turn over in peripheral blood [16], raising questions about the mechanisms underlying their long‐term maintenance. Using BM from nine healthy donors and five individuals with multiple sclerosis, alongside blood from healthy donors, the authors investigated the role of the BM site in maintenance of both FOXP3^+^ and FOXP3^−^ regulatory CD4^+^ T cells.

Both regulatory subsets were found to be enriched within the CD69^+^ Ki67^−^ compartment of the BM. The authors also analyzed expression of granzyme K and granzyme B, reflective of memory and effector potential, respectively [17, 18]. Interestingly, they found that Tr1 were strongly enriched in GzmK^+^, but not GzmB^+^, T cells, suggesting that they may be established as a quiescent and long‐lived resident population within the CD69^+^ fraction of the BM. Notably, Schneider Revueltas et al. [1] also identified a CD4^+^ T cell cluster (Cluster 4; “T‐PD‐1”) with a Tr1‐like transcriptional signature, with GZMK expression enriched in CD69^+^ CD4^+^ BM cells relative to their CD69^−^ counterparts. These parallel findings suggest that the preferential inclusion of Tr1 among the CD69+ subset in the BM can be detected across individuals and using different experimental assessments.

Furthermore, unlike conventional memory T cells that rely on IL‐7, these BM Tr1‐like cells expressed low levels of IL‐7Ralpha but high levels of CD122 (IL‐2/15Rbeta), and their in vitro survival was rescued by IL‐15. In a previous issue of EJI, Pascutti et al. demonstrated that a substantial proportion of CD69^+^ CD8^+^ BM TRM in mice require IL‐15 for their maintenance [9]. This new work by Pulvirenti et al. [12] extends this IL‐15 narrative to Tr1‐like cells, suggesting that IL‐15 availability may be an important feature in supporting BM TRM populations. Tr1 cells express the transcription factor Eomes that instructs upregulation of CD122 expression [19, 20], indicating that Tr1 cells employ this pathway for access to IL‐15 in the BM. Overall, this work provides insight into how a specialized regulatory population may also be maintained long‐term in the BM, contributing to our broader understanding of memory T cell diversity across tissues.

## 4 Concluding Remarks and Implications

Together, the studies by Schneider Revueltas et al. [1] and Pulvirenti et al. [12] help to challenge our ideas surrounding BM T cell residency. Schneider Revueltas et al. [1] reveal that CD69^−^ memory T cells in the BM are distinct from circulating cells, but also from CD69^+^ BM subsets. These findings argue against the notion that CD69^−^ BM cells are an integrated part of the circulating T cell pool that is trafficking through the BM. However, they also raise the possibility of compartmentalization within the resident pool itself. If both CD69^−^ and CD69^+^ BM memory populations are indeed resident, this would imply further specialization among BM‐resident T cell subsets, potentially linked to differences in antigen history, function, or maintenance cues. In contrast, Pulvirenti et al. [12], focusing on BM T cells with regulatory phenotypes, identify CD69^+^ EOMES^+^ GzmK^+^ Tr1‐like cells selectively maintained in the BM through IL‐15, distinguishing them from IL‐7‐dependent memory cells. Together, these findings highlight both the functional diversity of resident T cell populations in the BM, as well as potential roles for cytokine availability in determining niche occupancy.

**FIGURE 1:**
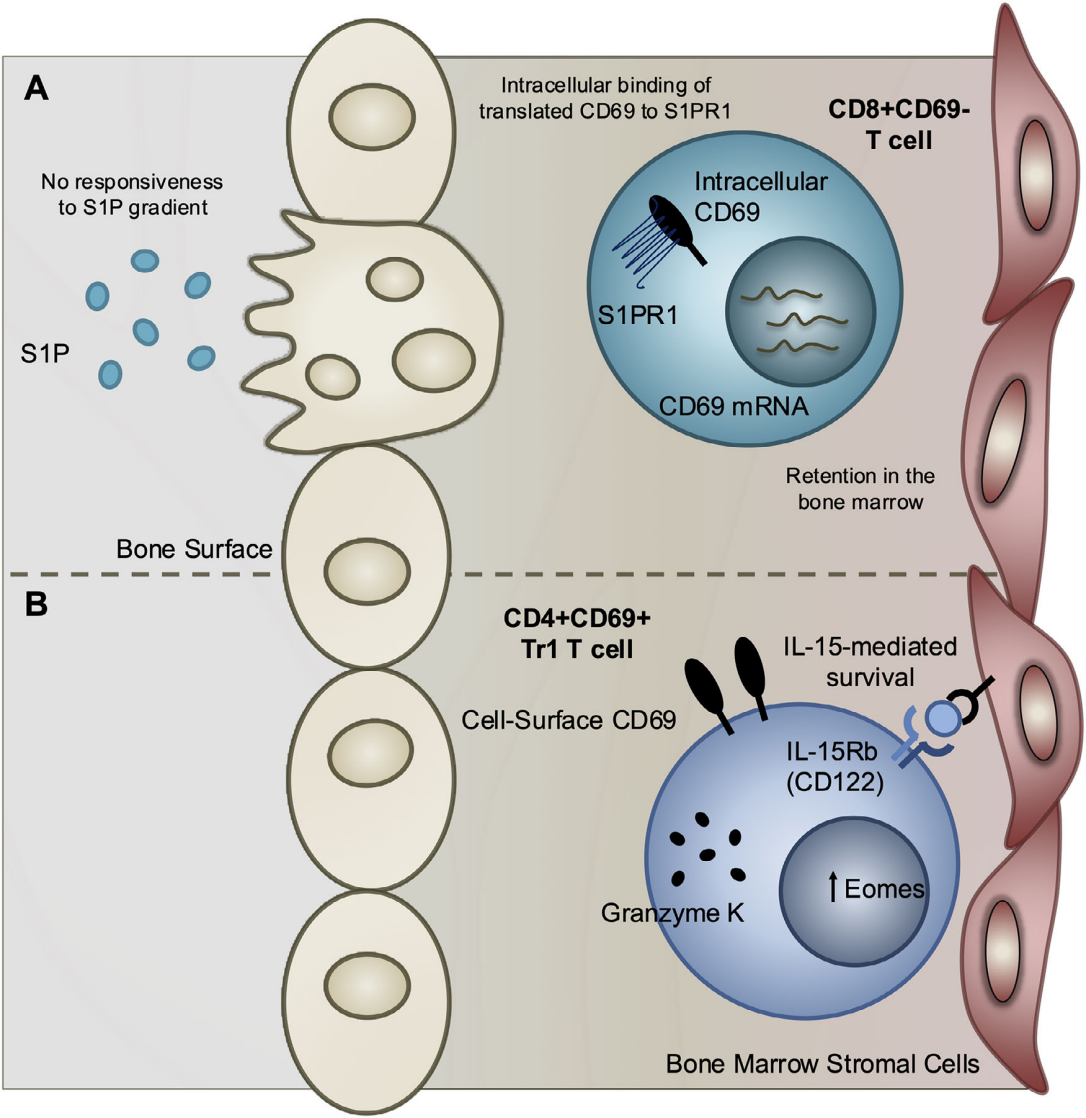
**Proposed mechanisms supporting distinct bone marrow resident T cell subsets**. (A) CD8+CD69^−^ memory T cells (Schneider Revueltas et al. [1]): Despite lacking surface CD69 expression, these cells transcribe CD69 and S1PR1, leading to intracellular CD69–S1PR1 binding that prevents responsiveness to sphingosine‐1‐phosphate (S1P) gradients to promote retention in the bone marrow niche. (B) Tr1‐like cells (Pulvirenti et al. [12]): CD69⁺ EOMES⁺ GzmK⁺ Tr1 CD4⁺ T cells are selectively maintained in the bone marrow through IL‐15‐mediated survival signals, in contrast to conventional IL‐7‐dependent memory T cells. Together, these proposed mechanisms illustrate complementary strategies by which diverse T cell subsets may reside long‐term within the bone marrow.

Although differing in scope, both studies reinforce the view of the BM as a compartmentalized immune niche capable of sustaining diverse T cell subsets through multiple, and perhaps unconventional, mechanisms. They expand the potential role of BM in T cell maintenance to include not only proinflammatory, but also regulatory populations, as evidenced by Tr1‐like cells with both suppressive and cytotoxic potential. This dual capacity raises important questions: How do distinct BM T cell subsets contribute to systemic versus local immune protection? What signals drive their differentiation, and can they be selectively expanded through vaccination or immunotherapy? Does BM residency confer functional advantages over circulating memory T cells, particularly in chronic infection, cancer, or aging? Addressing these questions will be essential to understanding the BM not merely as a storage site, but as an active, adaptable tissue that preserves a diverse and functionally competent pool of long‐lived T cells. By broadening perspectives on residency and niche specialization, these studies provide a framework for future efforts to harness BM immunity in health and disease (Figure 1).

## Conflicts of Interest

The authors declare no conflicts of interest.

## Data Availability

The authors has nothing to report.
